# The future of cell-free synthetic biology

**DOI:** 10.1016/j.biotno.2024.11.001

**Published:** 2024-11-09

**Authors:** Yuan Lu

**Affiliations:** Department of Chemical Engineering, Tsinghua University, Beijing 100084, China; Key Laboratory of Industrial Biocatalysis, Ministry of Education, Tsinghua University, Beijing 100084, China

**Keywords:** Cell-free synthetic biology, *In vitro* synthetic biology, Cell-free protein synthesis, Cell-free gene expression, Cell-free transcription-translation, Future perspectives

## Abstract

Cell-free synthetic biology aims at the targeted replication, design, and modification of life processes in open systems by breaking free of constraints such as cell membrane barriers and living cell growth. The beginnings of this systematized technology, which took place in the last century, were used to explore the secrets of life. Currently, with its easy integration with other technologies or disciplines, cell-free synthetic biology is developing into a powerful and effective means of understanding, exploiting, and extending the structure and function of natural living systems. Cell-free synthesis technology has been used in basic and applied research such as metabolic prototyping, artificial cell construction, nucleic acid engineering, protein engineering, etc. Worldwide experts shared their views and opinions on the future development of cell-free synthetic biology, as illustrated in this paper. With the integration of current popular technologies such as artificial intelligence and electronics, cell-free synthetic biology will play an increasingly important role in fields such as life exploration, intelligent manufacturing, human health, new energy, and sustainable environmental development.

## 1 Introduction

Cell-free synthetic biology, also known as *in vitro* synthetic biology, solution biochemistry, etc. The core concept is to achieve life processes such as transcription, translation, catalysis, metabolism, and signal transduction in an *in vitro* cell-free system. The key technologies are called cell-free protein synthesis, cell-free transcription-translation, cell-free gene expression, etc. Compared with traditional cellular systems, cell-free systems have outstanding characteristics such as openness and controllability, which can quickly and flexibly design and modify life processes from bottom to top, achieving a deeper understanding and wider application of natural life systems with great engineering freedom. In addition, due to the absence of cell membranes and constraints on cell growth, cell-free systems can be freely fused with non-living molecules, materials, equipment, etc., greatly expanding the functionality and application scope of natural living systems.

This cell-free synthetic biology research field has gone through roughly three golden periods. The first period was in the 1960s, focusing on basic research, such as the exploration of the transcriptional translation mechanism of the biological central dogma, which was the embryonic form of cell-free biosynthesis systems. The second period was at the beginning of this century. Cell-free biosynthesis systems were expanded to engineering and industrial applications, mainly focusing on synthesizing difficult-to-express proteins. The third period is the present 2020s. With the development of new technologies such as biology, chemistry, materials, and computers, as well as the new demands of society for health, energy, resources, environment, and other aspects, cell-free biosynthesis systems are increasingly being used in challenging basic and applied research.

At present, cell-free synthetic biology has entered a period of rapid development and application. Currently, the cell-free biosynthesis platform includes three major systems: multi-enzyme catalyzed synthesis system, component-specific transcription/translation system, and crude extract-based transcription/translation system. The development of its chassis has originated from diverse model or non-model prokaryotic or eukaryotic cells. At the basic research level, cell-free systems are used to explore dark matter in living systems, design system prototypes, construct artificial cells, and perform other fascinating research. At the application research level, cell-free systems are used for functional molecule screening, nucleic acid synthesis, complex protein synthesis, and non-natural biological molecule synthesis. Furthermore, by integrating with the evolving novel technologies, cell-free synthetic biology continues to radiate new vitality for technology development.

The future development of cell-free synthetic biology is a common concern for synthetic biologists. Experts worldwide in this field were invited to express their opinions and perspectives. Based on their own research content and understanding of the field, experts shared their views, ideas, challenges, and prospects. The specific information is shown below.

## 2 Opinions from worldwide experts

**Allen Liu**.

University of Michigan

*allenliu@umich.edu*.

The ability to construct programmable synthetic cell systems from the bottom up — with precise input-output relationships — promises transformative impacts on human health, biotechnology, and the environment. These systems will leverage the significant progress made over the past decades in cell-free protein expression, DNA/RNA-based gene circuits, and protein biochemistry. As the field advances toward creating use-inspired synthetic cell systems to address real-world problems, it must overcome three critical challenges. First, the ability to reliably and functionally reconstitute membrane proteins will be critical for endowing synthetic cells with environmental sensing capabilities. Second, ensuring a sustainable metabolism to provide energy to fuel protein synthesis will be a major hurdle. Third, integrating different functional modules will significantly expand the versatility of synthetic cells. From a fundamental science perspective, there are a lot of opportunities to investigate spatial and temporal control of cytoskeleton assemblies of enzyme activities. This could help recreate complex features of cellular shape changes and compartmentalized signaling, at both the single-cell level and in multi-cellular assemblies.

**Arnold Driessen**.

University of Groningen

*a.j.m.driessen@rug.nl*.

One of the main challenges in biology is to construct life bottom-up from individual components ideally programmed by a minimized genome. As an approximation, synthetic cell research depends on *in vitro* transcription/translation systems to synthesize the protein components of the cell. These can be either crude cell lysates or built from purified components such as the PURE system that includes, amongst others, essential components such as ribosomes that so far resisted *in vitro* reconstitution. Despite the general versatility of these systems, some major breakthroughs will be required for cell-free systems to support the autonomous replicating and dividing of synthetic cells. While cell-free synthesis of biosynthetic pathways is possible, the sheer number of genes to be translated in sufficient numbers for a synthetic cell still provides a formidable challenge. Further, efficient methods are needed to trap cell-free systems into giant unilamellar vesicles while maintaining the permeability barrier of the membrane. Finally, specifically for membrane proteins, their synthesis and membrane insertion should be coordinated to prevent protein aggregation and misfolding and, hence, poor productivity of the synthetic cell.

**Atsushi Ogawa**.

Ehime University

*ogawa.atsushi.mf@ehime-u.ac.jp*.

As the name implies, cell-free synthetic biology seeks to create living cells from cell-free components. While it is academically important to create artificial cells that are identical or similar to living cells found in nature, creating artificial cells with functions that surpass those of natural cells for the benefit of society would also be a major step forward in this field. Many challenges must be overcome to achieve these goals, and one of the most pressing is how to assemble well-controlled and complex genetic circuits. Our research subject, cell-free artificial riboswitches, will help to address this challenge.

**Bo Zheng**.

Shenzhen Bay Laboratory

*bozheng@szbl.ac.cn*.

The research of *in vitro* reconstitution of ribosomes has made significant progress in recent years. In the near future, we expect to witness a cell-free transcription-translation (TX-TL) system that is capable of self-replication. However, the construction of self-replicating synthetic cells based upon the self-replicating cell-free TX-TL system still faces mounting challenges, such as the complexity of membrane regulation. Membraneless porous particles enriched with cell-free TX-TL system could circumvent the challenges and be the stepping stones to the first self-replicating synthetic cells.

**Dong-Myung Kim**.

Chungnam National University

*dmkim@cnu.ac.kr*.

Cell-free synthetic biology will play transformative roles in personalized medicine and diagnostics. Cell-free protein synthesis enables rapid, customizable protein production, bypassing traditional cell cultivation to meet the growing demand for individualized therapeutic solutions. This flexibility is essential for producing tailored biologics on demand, aligning with the shift toward patient-specific treatments. In diagnostics, the simplicity and modularity of cell-free synthesis allow for the generation of reporter proteins in response to specific biomarkers, such as proteins and nucleic acids, enhancing multi-target detection capabilities. Its portability and simplicity also make it ideal for point-of-care applications, especially in resource-limited settings. As related technology advances, cell-free synthesis is poised to redefine biomanufacturing and diagnostics, supporting next-generation healthcare with greater efficiency and adaptability.

**Fernando López Gallego**.

CIC biomaGUNE

*flopez@cicbiomagune.es*.

Assembling isolated enzymes, either in their pure or crude formulation, into biosynthetic pathways of a certain complexity (including more than 4 enzymes) has given rise to a new discipline: cell-free synthetic biology, also coined as synthetic biochemistry or systems biocatalysis. Most existing examples are limited to free enzymes, leading to systems with low robustness that are difficult to transfer across different reactor configurations for further intensification. The rational co-immobilization of multi-enzyme systems on solid porous materials is opening a new avenue to intensify and scale up both natural and artificial biosynthetic pathways. Yet, their implementation in flow reactors for their continuous operation is scarce. Hence, new advances in enzyme immobilization technology and in the analytics for characterizing multi-functional heterogeneous biocatalysts are demanded to increase the complexity of biosynthetic pathways in the solid phase. Heterogeneous cell-free synthetic biology should aim to match the complexity achieved by cell-free systems in solution, where up to 54 enzymes have been assembled, while also enhancing product titers and enzyme and cofactor turnover numbers.

**Filippo Caschera**.

Consultant

*filippocaschera@gmail.com*.

Cell-Free Protein Synthesis (CFPS) will supercharge drug discovery, improving human health. CFPS is an easily accessible platform for expressing and prototyping proteins at unprecedented speed and throughput with robotic workstations and microfluidic systems. New macromolecules will be designed using Generative AI or Active Learning – Assisted Directed Evolution. The Design-Build-Test-Learn cycle will enable the discovery of antibodies and antigens conjugates to develop more efficient cancer therapies and fight infectious diseases. New paradigms will be advanced towards antimicrobial activity by engineering new peptides and phage therapies. Moreover, CPFS is the favourable method to produce membrane proteins such as GPCRs, which represent the major target for drug development. Consequently, Cell-Free Protein Synthesis will be a platform used in every pharmaceutical and biotechnology company worldwide.

**Jamin Koo**.

Hongik University

*jaminkoo@hongik.ac.kr*.

Future trends in cell-free synthetic biology will revolutionize the synthesis of novel metalloproteins with tailored shapes and structures. By bypassing the complexities of living cells, cell-free systems enable precise control over protein design, including the incorporation of non-natural amino acids and cofactors. This will facilitate the creation of metalloproteins with enhanced or entirely new functions, expanding their applications in catalysis, bioremediation, and therapeutic development. Recent advances, such as the use of cell-free systems to synthesize complex metalloproteins, suggest a promising path toward the rapid production of custom-designed biomolecules for diverse industries.

**Jian Li**.

ShanghaiTech University

*lijian@shanghaitech.edu.cn*.

Cell-free synthetic biology has evolved from a fundamental research tool into a versatile platform with great potential. Historically, cell-free systems played a pivotal role in deciphering genetic codes. Looking ahead, I envision cell-free systems emerging as powerful biotechnological tools for biomanufacturing and the bioeconomy. My research focuses on the cell-free biosynthesis of complex pharmaceutical natural products, and I believe these systems will provide efficient pathways for natural product biosynthesis, significantly accelerating discovery and development processes. The future of cell-free synthetic biology is promising, poised to drive innovations that will transform the field of biotechnology.

**Kei Fujiwara**.

Keio University

*fujiwara@bio.keio.ac.jp*.

Advances in cell-free technology are bridging the gap between purified systems and living cells. This progress enables more detailed emulation of cellular processes and will clarify the fundamental differences between *in vitro* and *in vivo* environments, which were often dismissed as artifacts. Consequently, we are gaining insights into the essential blueprint of life, which holds the potential for a deeper understanding of biological systems. This achievement transforms molecular biology from a field that observes and identifies components of life into one that literally assembles and understands biology at the molecular level.

**Lei Kai**.

Jiangsu Normal University

*lkai@jsnu.edu.cn*.

The exciting pursuit of building a cell through bottom-up approaches has captured the attention of multidisciplinary communities, emerging as a distinct research field. Over the past two decades, significant advancements have been made in constructing synthetic cellular mimics characterized by compartmentalization, metabolism, replication, and emergent properties such as evolution, responsiveness, and movement. However, challenges remain in developing key technologies to integrate these components effectively. This includes refining tunable compartmentalization techniques and creating long-lasting, autonomous cell-free expression systems. Continuous improvement in encapsulation efficiency and system homogeneity, even through microfluidic platforms, is essential. Additionally, enhancing ribosome efficiency within cell-free systems, especially toward constructing a fully synthetic ribosome, remains vital. In addition, other enabling technologies, such as DNA synthesis, imaging technologies, automation, computational modeling as well as AI, would greatly accelerate such progress. Finally, large-scale collaboration across laboratories, consortia, and continents is necessary to enable seamless communication and efficient technology transfer, ultimately paving the way toward the creation of a minimal synthetic cell.

**Mark Styczynski**.

Georgia Institute of Technology

*mark.styczynski@chbe.gatech.edu*.

Cell-free synthetic biology has tremendous potential for broad scientific and societal impact. As costs come down and productivities go up, cell-free synthetic biology is destined for increasingly broad industrial applications that could substantially impact biomanufacturing even if it cannot displace several well-established biomanufacturing strategies. In the intervening time, small-volume applications like diagnostics are likely to be the main vehicle for impact, from biomedical to environmental applications. As these applications make their way through regulatory approval and adoption, they will be the first ways that we put the benefits of cell-free approaches into the hands of everyone.

**Shaobin Guo**.

Fuzhou University

*sguo@fzu.edu.cn*.

As this year's Nobel Prize in Chemistry recognizes breakthroughs in rational protein design and AI-driven structure prediction, cell-free systems will likely see increased integration with artificial intelligence for protein design and optimization, enabling faster iteration and high-throughput screening. Besides, despite being in the early stages, synthetic cells based on cell-free systems are emerging as powerful platforms for mimicking cellular functions without the complexity of living cells. By enabling precise control over reaction conditions and the components involved, synthetic cells offer a streamlined environment for the development of complex gene circuits, allowing rapid testing and iteration of circuit designs.

**Yoshihiro Shimizu**.

RIKEN

*yshimizu@riken.jp*.

We're strongly interested in synthetic cell projects that aim to answer fundamental questions: what is life? Recently, we have been studying crucial modifications on tRNAs, beneficial for the tRNA reconstitution, as well as how to assemble active ribosomes from their molecular parts, both performed in cell-free systems. We are also interested in synthesizing beneficial products with cell-free systems to solve biological questions in other research fields. We have discovered that cell-free synthetic biology can contribute to quantitative proteome analysis, and we are promoting research in such a direction for the development of medicine and analysis of the molecular status in higher organisms.

## 3 Conclusion

The perspectives shared by these fourteen experts worldwide on the future development of cell-free synthetic biology would inspire students, teachers, researchers, government decision-makers, and business personnel engaged in synthetic biology or biotechnology-related fields. The announcement of this year's Nobel Prize has once again pushed the application of artificial intelligence in the field of biology to another climax. Artificial intelligence tools can accelerate the design of biomolecules, but there are two important challenges that need to be addressed after design: how to synthesize them through experiments, and how to quickly screen for such a large sample size. In this context, cell-free synthetic biology will leverage its unique and significant advantages to address the dual issues of synthesis and screening. With the development and demand of important fields such as new energy, materials science, bioelectronics, neuroscience, and carbon neutrality, cell-free synthetic biology will also play its unparalleled advantages and roles.

## Declaration of competing interest

The author is an Editor-in-Chief for Biotechnology Notes and was not involved in the editorial review or the decision to publish this article.

